# Estimating the burden of care home gastroenteritis outbreaks in England, 2014–2016

**DOI:** 10.1186/s12879-018-3642-3

**Published:** 2019-01-05

**Authors:** Thomas Inns, Helen E. Clough, John P. Harris, Roberto Vivancos, Natalie Adams, Sarah J. O’Brien

**Affiliations:** 1 Institute of Psychology, Health and Society, University of Liverpool, Liverpool, UK; 2 NIHR Health Protection Research Unit in Gastrointestinal Infections, University of Liverpool, Liverpool, UK; 3 National Infection Service, Public Health England, London, UK; 4 NIHR Health Protection Research Unit in Emerging and Zoonotic Infections, University of Liverpool, Liverpool, UK

**Keywords:** Norovirus, Gastroenteritis: Care homes, Outbreaks, Surveillance

## Abstract

**Background:**

Outbreaks of infectious gastroenteritis in care homes are common, with norovirus a frequent cause. In England there is no co-ordinated national surveillance system. We aimed to estimate the burden of these outbreaks.

**Methods:**

Using a generalised linear mixed effects regression model we described the relationship between the observed number of care home outbreaks and covariates. Estimated model parameters were used to infer uplift in the number of outbreaks expected if all areas were subjected to enhanced surveillance. From this we then estimated the total burden of care home gastroenteritis outbreaks in this period.

**Results:**

We estimated a total of 14,146 care home gastroenteritis outbreaks in England during 2014–2016; this is 47% higher than the reported total and a rate of 32.4 outbreaks per 100 care homes per year. The median number of outbreaks from the model estimates was 31 (IQR 20–46) compared to 19 (IQR 12–34) reported from routine surveillance.

**Conclusions:**

This estimated care home gastroenteritis burden in England indicates that current surveillance substantially underestimates the number of outbreaks, by almost half. Improving this surveillance could provide better epidemiological knowledge of the burden of norovirus to inform public health policy, particularly with the advent of norovirus vaccines.

**Electronic supplementary material:**

The online version of this article (10.1186/s12879-018-3642-3) contains supplementary material, which is available to authorized users.

## Background

Residential care homes for the elderly provide an ideal environment for acquisition and spread of infection [1]. Outbreaks of infectious gastroenteritis in care homes are common, with 16.8 outbreaks per 100 care homes per year being reported from a study in Australia [2]. Norovirus is the pathogen which has been reported as being the most frequent cause of care home infectious gastroenteritis outbreaks [3]. Norovirus is estimated to be responsible for 10–20% of gastroenteritis hospitalisations in older adults [4] and has been associated with excess mortality in the elderly [5]. There are few surveillance systems to detect norovirus disease in community settings [6]. There are surveillance systems which capture information on infectious gastroenteritis outbreaks in care homes in France [7] and Australia [8].

In England, information on general outbreaks of infectious gastroenteritis in care homes has been collected since 1992 [9]. Since 2010, the Care Quality Commission (CQC) has required care homes in England to report outbreaks of infectious gastroenteritis to Public Health England (PHE) [10]. Despite this, there is no co-ordinated national surveillance system to collect this information; in most of England, this information is captured locally by PHE using a health protection case management tool [11]. However, in certain areas of England there are enhanced surveillance systems that capture more detailed information on care home gastroenteritis outbreaks [12, 13].

Given the lack of a dedicated surveillance system, there is no routine way of calculating the burden of care home gastroenteritis outbreaks. Estimating the magnitude of this burden is important as it quantifies the direct impact upon the facilities and can also be used to infer indirect impacts on hospitals, given that patients are often transferred between care homes and hospitals. In this research we used a modelling approach to estimate the total burden of care home gastroenteritis outbreaks in England, adjusted for under-reporting. Comparable approaches have previously been used to estimate the under-reporting of norovirus illness in the community [14].

## Methods

### Study design

In this analysis we used an ecological study design with the local authority in England as the unit of analysis. Data were aggregated at local authority level, for the period 1 January 2014 to 31 December 2016 (the study period). The number of care home gastroenteritis outbreaks in each area in the study time period was the primary outcome. From this we calculated the reported rate of care home gastroenteritis outbreaks per 100 care homes per year.

### Study definitions

We defined a care home as a facility providing long-term residential care, with or without nursing care. An outbreak was defined as either “two or more cases of gastrointestinal infection occurring around the same time, in residents or their carers” or “an increase in the number of cases above the number normally observed” [15]. Routine surveillance is defined as a system that captures basic information on an outbreak (care home name, date of outbreak, number of cases). Enhanced surveillance is defined as a system that captures more detailed information than routine surveillance (eg. outbreak duration, population denominator, pathogen isolated). Both enhanced and routine surveillance are passive surveillance systems.

### Data sources

In England care homes have a legal requirement to register with, and be inspected by, the CQC in accordance with Schedule 1 of The Health and Social Care Act 2008 (Regulated Activities) Regulations 2014. The CQC database of registered care homes [16] was queried to obtain the number of care homes by local authority. The relevant PHE surveillance systems were queried to obtain the number of care home outbreaks in each local authority reported during the study period.

The Office for National Statistics provides population data for England. For each local authority, the following data were obtained: the total population, the proportion of the population under the age of 5 years and the proportion of the population over 65 years old [17]. We included the proportion of the population under 5 in our analysis as rates of norovirus infection are significantly higher in this group compared to those in other age groups [18]. All public hospital laboratories in England report data to the Second Generation Surveillance System (SGSS) [19]. From SGSS we obtained the number of laboratory confirmed norovirus cases in the study period by local authority. In England, the Department for Education maintain a database of all schools. From this, we obtained the number of primary schools (for children aged 4–11) in each local authority [20]. We included primary schools as an explanatory variable in our model because schools are the community institution most affected by norovirus outbreaks besides care homes [21].

### Statistical methods

We described the data by calculating the rate of reported care home gastroenteritis outbreaks per 100 care homes per year for each local authority. We calculated this rate for the whole of England, along with the total number of reported outbreaks. We used hexagonal cartograms of local authorities in England to represent graphically the spatial variation in reported outbreak rate [22]. We used *t*-tests to compare the values of each explanatory variable for local authorities with routine surveillance to those with enhanced surveillance.

We used a generalised linear mixed model to describe the relationship between the number of outbreaks per local authority and a range of explanatory variables. The outcome variable for local authority *i* was the number of outbreaks in local authority *i*. The negative binomial family was chosen over the Poisson family to account for the fact that there was more variation in the count data than could be explained by the simpler Poisson distribution-based GLM. Random region-level intercepts were included to accommodate geographical variation and intrinsic but unmeasured differences between PHE regions [23].In this analysis we assumed that ascertainment of outbreaks was more complete in areas with enhanced surveillance (which collect more detailed information).

The explanatory variables selected a priori were: number of care homes, area population, proportion of the population under the age of 5 years, proportion of the population over the age of 65 years, number of laboratory confirmed norovirus cases, number of primary schools in the local authority. These were analysed as continuous variables. A binary variable was used to indicate whether a region was subject to enhanced surveillance or not. Where necessary, explanatory variables were rescaled to ensure model convergence. We then used the model together with a simulation-based approach to estimate what the number of outbreaks in each local authority might be if all local authorities in England had an enhanced surveillance system. We conducted these analyses using R [24], using the lme4 package for the regression model [25]. We undertook a sensitivity analysis to assess the effect of influential observations.

Our chosen model estimated the association between each explanatory variable and the number of gastroenteritis outbreaks. For area *i* the predicted count was simulated as a random realisation from a negative binomial distribution with shape parameter λ and mean μ set as the fitted value for area *i* which has been obtained directly using estimated parameters from the model based upon the original data but recoding all areas as if they were enhanced (enhanced = 1). Since sampling variation causes this number to vary each time it is simulated the whole process was repeated 10,000 times and these values were used to estimate the true number of outbreaks across England with an empirical 95% Confidence Interval. Model estimates were combined with recent study data on the characteristics of acute gastroenteritis outbreaks in care homes [12] to quantify the number of cases linked to these outbreaks. The statistical specification of this model is shown in Additional file 1.

### Results

**Fig. 1:**
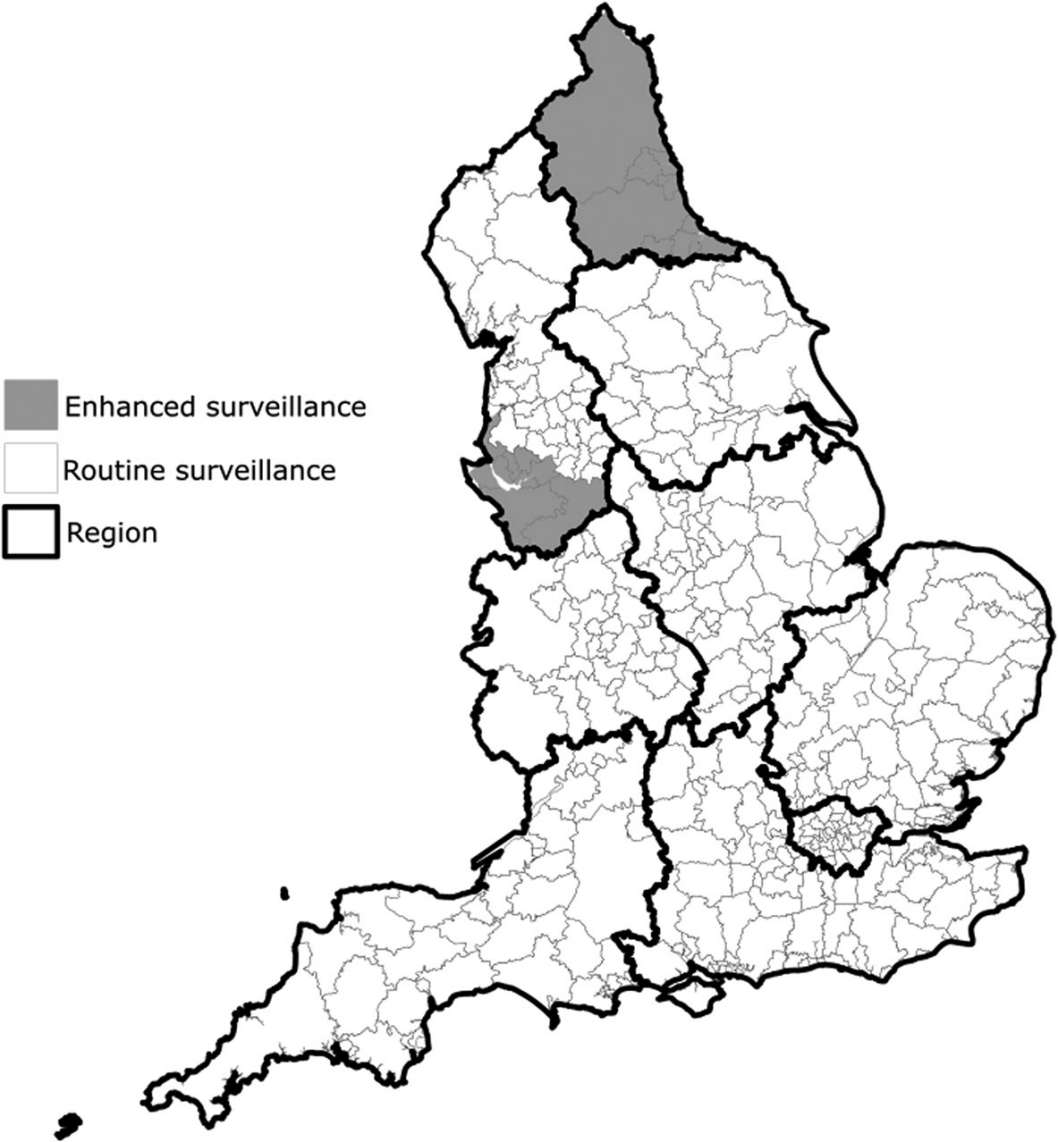
Map showing care home gastroenteritis surveillance system and PHE region, for each local authority (n = 326), England, 2014–2016

There are 326 local authorities in England of which, twenty one reported to an enhanced surveillance system. The geographical location of the local authorities with routine and enhanced surveillance is shown in Fig. 1. During the study period, there were 9594 gastroenteritis outbreaks in 14,229 care homes. A summary for each of the study variables, comparing routine and enhanced areas, is provided in Table 1 below.

**Table 1:** Summary characteristics for study variables, by surveillance type, for all local authority (n = 326), England, 2014–16

	Routine (*n* = 305)			Enhanced (*n* = 21)			
Variable	Median	25p	75p	Median	25p	75p	*p* value
Number of outbreaks	19	12	31	69	42	93	< 0.0001
Number of care homes	36	25	54	58	35	80	0.0037
Number of lab confirmed norovirus cases	31	13	64	28	15	48	0.6246
Total population	128,467	96,956	197,657	203,307	147,915	316,002	0.0030
Proportion under 5	0.06	0.05	0.07	0.06	0.06	0.06	0.5390
Proportion over 65	0.18	0.15	0.22	0.18	0.16	0.20	0.8626
Number of primary schools	54	40	73	75	54	106	0.0014

The number of laboratory confirmed norovirus cases (*p* = 0.6246), proportion of the population under 5 (*p* = 0.5390) and proportion of the population over 65 (*p* = 0.8626) were not significantly different between local authorities with routine and enhanced surveillance. Local authorities with enhanced surveillance had a significantly higher total population (*p* = 0.0030), greater number of reported outbreaks (*p* < 0.0001), greater number of care homes (*p* = 0.0037) and greater number of primary schools (*p* = 0.0014).

**Fig. 2:**
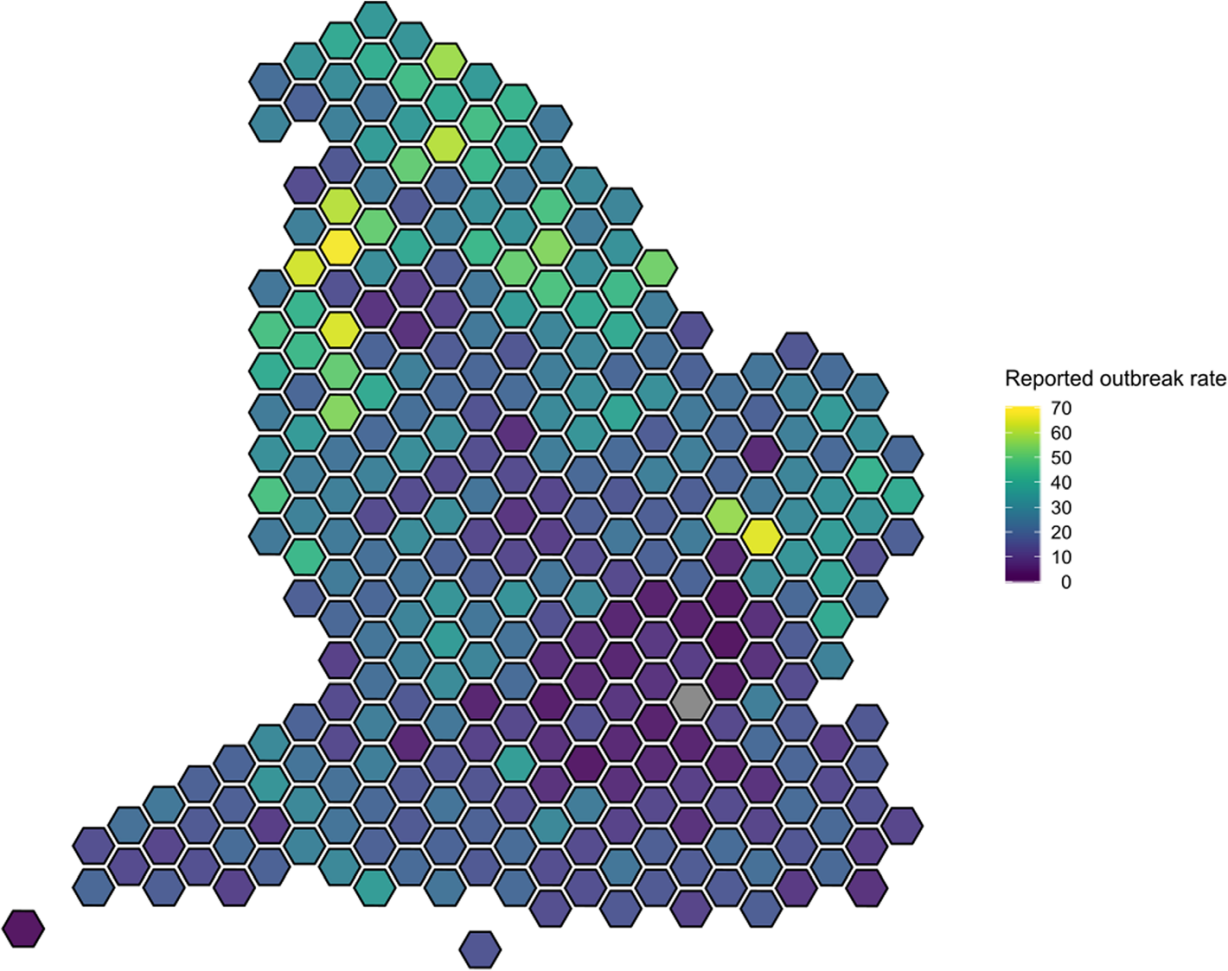
Hexagonal cartogram showing reported outbreak rate per 100 care homes per year, for each local authority (*n* = 326), England, 2014–2016

Over the three year study period, 22.48 outbreaks per 100 care homes per year were reported. The median rate was 20.37 outbreaks per 100 care homes per year (Interquartile range (IQR) 12.79–29.29 outbreaks per 100 care homes per year). The mean rate in the enhanced area was 39.67 (IQR 33.33–45.83), significantly higher than the mean rate of 21.40 (IQR 12.39–27.53) observed in the local authorities with routine surveillance (*p* < 0.0001). There is substantial geographical variation in the reported rate of gastroenteritis outbreaks in care homes (Fig. 2a).

The results of the negative binomial regression model with random effects are shown in Table 2. Simultaneously adjusting for all variables in the model, the variable most strongly associated with the outcome was the number of care homes (coefficient = 1.96, *p* = < 0.001). The other variables significantly associated with the outcome were the number of laboratory confirmed norovirus cases (coefficient = 1.08, *p* = 0.012) and the number of primary schools (coefficient = 1.15, *p* = 0.035). There are nine regions, these were included in the model as a random effect; intercepts varied from the lowest in London (− 1.217) to the highest in Yorkshire and Humber (0.578). In the sensitivity analysis without influential observations there were no changes to the direction, magnitude or significance of variable estimates from the negative binomial regression model with random effects.

**Table 2:** Results of negative binomial regression model with random effects (n = 326)

Variable	Coefficient	95% CI	*p*
Fixed effects			
Enhanced surveillance	1.54	1.16–2.03	0.003
Number of care homes	1.96	1.74–2.21	< 0.001
Number of laboratory confirmed norovirus cases	1.08	1.02–1.14	0.012
Total population	0.97	0.82–1.14	0.668
Proportion under 5	0.97	0.89–1.06	0.530
Proportion over 65	1.01	0.91–1.11	0.874
Number of primary schools	1.15	1.01–1.31	0.035
Random part (Region)	Intercept		
East Midlands	−0.021		
East of England	0.109		
London	−1.217		
North East	0.289		
North West	0.281		
South East	−0.181		
South West	−0.117		
West Midlands	0.285		
Yorkshire and Humber	0.578		

**Fig. 3:**
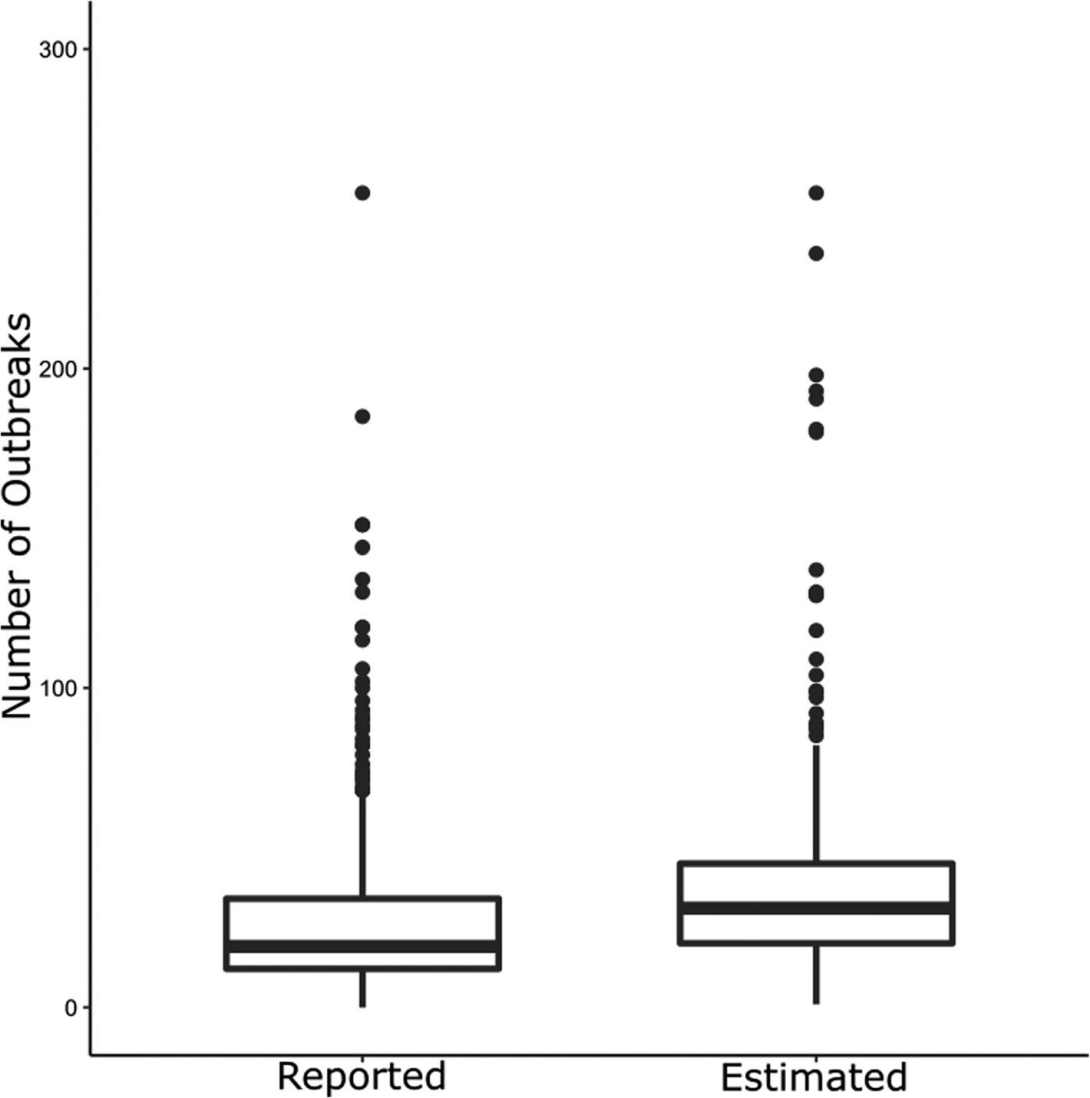
Box plot showing the distribution of care homes outbreaks for local authorities (n = 326), comparing reported count to the estimated count, England, 2014–2016

From this model, we estimate that there were a total of 14,146 (95% Confidence Interval 13,372 – 14,975) care home gastroenteritis outbreaks in England from 2014 to 2016. This is 4552 (47%) greater than the reported number and equates to a rate of 32.4 outbreaks per 100 care homes per year. The distribution of reported outbreaks is compared to the estimated numbers in Fig. 3 below. The median number of reported outbreaks was 19 (IQR 12–34), compared to 31 (IQR 20–46) from the estimated data.

A recent study [12] reported that; the median number of residents in a care home was 34, the median number of staff was 36; and that the median acute gastroenteritis attack rate was 30% in residents and 6% in staff. Based on these data, we hypothesise that in the region of 174,845 cases (144,289 residents and 30,556 staff) might have been affected in total.

## Discussion

From our analysis, we estimate that there were a total of 14,146 care home gastroenteritis outbreaks in England during this period, a 47% increase on the reported total of 9594 outbreaks. This is the first estimate of the total care home gastroenteritis burden of infection in England and translates to a rate of 32.4 outbreaks per 100 care homes per year in England. This is important as acute gastroenteritis, particularly norovirus gastroenteritis, is a common cause of morbidity, especially in the elderly [5]. Care home gastroenteritis outbreaks should largely be preventable with good infection prevention and control [26]. This study provides evidence to show that we are currently underestimating this burden which not only has direct impacts on residents and staff at the facilities, but also wider impacts on hospitals though delayed discharges and importation of cases which can cause outbreaks and subsequent bed closures [27].

For international comparison, this estimated rate of 32.4 outbreaks per 100 care homes per year in England is higher than the reported rates from Australia (16.8 [95% confidence interval, 12.4–22.7] outbreaks per 100 care homes per year) [2], and far higher than the reported rate from France (4.6–5.5 outbreaks per 100 care homes per year) [7]. These comparison data have not been adjusted for under-reporting in the way we have used in this paper; were this to happen, it could be that the estimated rates in these countries would be closer to that estimated from our model for England. Other factors which could have been associated with differences in reported outbreak rates include: different populations at risk, different structural, organisational or infection control arrangements, or different levels or types of circulating pathogens during the study period.

In this study our definition of gastroenteritis was not pathogen-specific. Norovirus is a common cause of acute gastroenteritis in care homes, with numerous introduction routes and risk factors for spread [28]. Norovirus incidence has a bimodal distribution and after children under 5, the elderly are the next most affected group [18]; in this group norovirus has been associated with mortality [5] and between 2014 and 2016 there were between 20 and 31 deaths directly attributed to norovirus infection those aged over 60 years which represents between 86 and 97% of norovirus-attributed deaths annually [29]. Data from the United States reported the number of norovirus outbreaks in care homes [30], but without robust denominator data on the number of facilities, it is not possible to compare outbreak rates. Norovirus has been estimated to cause between 48% [9] and 73% [7] of care home gastroenteritis outbreaks, but there is no suitable contemporary data from England which could be used to estimate the proportion of this outbreak burden which is caused by norovirus. If this were available, it would be a logical extension of this work to apply that to this estimate, and use this in conjunction with other work [31] to estimate the burden of norovirus gastroenteritis in the community. Considering the potential of norovirus vaccines currently in development, this data on the burden of norovirus in the community could be combined with estimates of the hospital burden of norovirus disease [32] to provide a baseline to assist vaccine policy-makers.

Gastrointestinal disease data collected in surveillance systems are frequently an underestimation of the underlying burden of illness [33]. The use of multiplication factors to adjust for under-reporting is a common approach, but there is a need for them to be well calibrated to each context [34]. In this analysis, we estimated that the burden was approximately 50% higher than the reported data. This indicates that there is substantial capacity to improve the surveillance configuration in England to effectively capture these outbreaks. It is also likely that even enhanced surveillance systems missed outbreaks because they were not reported by the care homes despite the legislation. A previous study [12] suggested that higher attack rates were associated with late reporting. Given the possibility that even enhanced systems missed some outbreaks, it is likely that our estimate of the outbreak burden is a conservative one.

In our model we observed a significant positive association between the number of primary schools in a local authority and the number of care home outbreaks. This would be expected as rates of norovirus infection are significantly higher in children compared to those in other age groups [18] and schools are commonly affected by norovirus outbreaks [21]. Therefore, an area with care home gastroenteritis outbreaks would also be expected to have school gastroenteritis due to pathogens circulating in the community. There is no comprehensive or reliable dataset of school gastroenteritis outbreaks in England, so the number of primary schools was included as a proxy for this information. This relationship between primary school and care homes, and its relevance to transmission of norovirus, should be considered when formulating potential vaccination strategies as and when a vaccine is available.

In this study we used a binary classification (routine/enhanced) of surveillance system which is likely to have been a crude measure of the effectiveness of these systems. In each area, a number of factors will affect the effectiveness of surveillance; the care home management, the engagement of community infection control staff, practices of local PHE staff amongst many reasons. For example, in the East of England during this period there was a surveillance system in place, but this system did not meet our definition of an enhanced system as this system did not collect additional information. This binary classification is therefore a limitation, but makes the analysis feasible given the available data. Additionally, this analysis was predicated on the assumption that outbreak ascertainment was greater in those areas with enhanced. The results of this study provide evidence to support this assumption, but further work is needed to understand the precise characteristics of enhanced systems that increase outbreak ascertainment, so that these can be adopted more widely.

Ideally we would like to have enhanced surveillance spread more evenly across regions as this represents a potential source of sampling bias. However the nature of surveillance systems means that areas which are and are not enhanced is beyond our control and is predetermined by external factors such as local structures, funding and research interests. Due to the retrospective observational nature of this study randomising the nature of surveillance systems was not possible and we have instead sought to control for biases and confounders using an appropriate generalised linear model-based methodology. We thus interpret estimates provided by our approaches fairly cautiously and acknowledge the inherent uncertainties.

Another potential limitation of the study is the ecological design which means that any inference from this analysis is restricted to the level of the local authority and therefore it is not possible to make any conclusions at the level of the individual care home. In our model we used random effects to provide information on PHE regions. This was intended to account for differential practice between PHE teams; these showed that accounting for other explanatory variables, some areas such as London had lower counts of care home gastroenteritis outbreaks than other regions such as Yorkshire and Humber.

## Conclusions

Our results indicate the current mixed surveillance approach to gastroenteritis outbreak surveillance in care homes in England is considerably underestimating the burden of infection. This translates to a substantial burden of infection on staff and residents or these institutions, along with indirect impacts on the wider healthcare system. To reduce this underestimation, we recommend that Public Health England work towards implementing a surveillance system to standardise the collection of these outbreak data. Linked to this work on the surveillance system, Public Health England should liaise with the CQC, community infection control staff and care home managers to communicate the importance of this form of surveillance. Comprehensive and timely surveillance of care home gastroenteritis outbreaks could improve public health practice by highlighting areas of effective infection control and providing an early warning of an intensive norovirus season which could inform hospital bed management.

## Additional file

Additional file 1: Summary of statistical model. A statistical description of the statistical model used in this study (DOCX 13 kb)

## Abbreviations

CQC: Care Quality Commission
IQR: Interquartile range
PHE: Public Health England
SGSS: Second Generation Surveillance System
