# Supplementary material for: Estimating the burden of care home gastroenteritis outbreaks in England, 2014–2016
Source: BMC Infect Dis. 2019 Jan 5;19:12. doi: 10.1186/s12879-018-3642-3 (PMC6321657; doi:10.1186/s12879-018-3642-3)
Supplement: Supplementary file 1 — Summary of statistical model. A statistical description of the statistical model used in this study (DOCX 13 kb) [file 12879_2018_3642_MOESM1_ESM.docx]

**Supplementary material**

The negative binomial mixed effects model is a model for count data which extends the Poisson mixed effects model. It is useful when data are over-dispersed in that it relaxes the Poisson model constraint that the conditional mean and variance within the model are the same (model dispersion parameter fixed at 1), and allows the variance to be greater than the mean, which is commonly what is required for over-dispersed data. Technically, the negative binomial model arises by first specifying a Poisson model for the observed data *Y_ij_* and then allowing the rate parameter $\mu$ within that model to follow a gamma distribution (Faraway, 2016). A region-level random effect, $U_{j}$ such that $U_{j}\sim N(0,\tau^{2})$ was incorporated in the model to reflect the fact that observations from the same region *j* may be more similar than those from different regions.

Let $Y_{ij}$ , *i* = 1,…, *n* be the count for individual (local authority) i in region *j*. Then conditional on random effects $U_{j}\sim N\left( 0,\tau^{2} \right)$, the *Y_i_*_j_ are independent negative binomial random variables

$$Y_{ij}\sim NegBin(\alpha, \mu_{ij})$$

such that

$$\mu_{ij}=E\left( Y_{ij}|U_{j} \right)=exp\left[ {x_{ij}}^{'}\boldsymbol{\beta}+U_{j} \right]$$

and for *p* independent explanatory variables, *β* is an unknown *p* x 1 vector of regression coefficients (See, for example, Booth *et al.* (2003)).

Booth, J. G., Casella, G., Friedl, H., & Hobert, J. P. (2003). Negative binomial loglinear mixed models. Statistical Modelling, 3(3), 179–191. <https://doi.org/10.1191/1471082X03st058oa>

Faraway, J. J. (2016). Extending the Linear Model with R: Generalized Linear, Mixed Effects and Nonparametric Regression Models, Second Edition (Chapman & Hall/CRC Texts in Statistical Science).
